# Effectiveness of a Constructed Wetland with Carbon Filtration in Reducing Pesticides Associated with Agricultural Runoff

**DOI:** 10.1007/s00244-021-00909-0

**Published:** 2022-01-05

**Authors:** Laura B. McCalla, Bryn M. Phillips, Brian S. Anderson, Jennifer P. Voorhees, Katie Siegler, Katherine R. Faulkenberry, Maurice C. Goodman, Xin Deng, Ron S. Tjeerdema

**Affiliations:** 1 Marine Pollution Studies Laboratory at Granite Canyon, Department of Environmental Toxicology, University of California, Davis, Monterey, CA USA; 2 Hopkins Marine Station, Stanford University, Pacific Grove, CA USA; 3 Surface Water Protection Program, California Department of Pesticide Regulation, Sacramento, CA USA

## Abstract

The Salinas Valley in Monterey County, California, USA, is a highly productive agricultural region. Irrigation runoff containing pesticides at concentrations toxic to aquatic organisms poses a threat to aquatic ecosystems within local watersheds. This study monitored the effectiveness of a constructed wetland treatment system with a granulated activated carbon (GAC) filter installation at reducing pesticide concentrations and associated toxicity to *Ceriodaphnia dubia*, *Hyalella azteca*, and *Chironomus dilutus*. The wetland was supplied with water pumped from an impaired agricultural and urban drainage. Across five monitoring trials, the integrated system’s average pesticide concentration reduction was 52%. The wetland channel and GAC filtration components individually provided significant treatment, and within each, pesticide solubility had a significant effect on changes in pesticide concentrations. The integrated treatment system also reduced nitrate by 61%, phosphate by 73%, and turbidity by 90%. Input water was significantly toxic to *C. dubia* and *H. azteca* in the first trial. Toxicity to *C. dubia* persisted throughout the system, whereas toxicity to *H. azteca* was removed by the channel, but there was residual toxicity post-GAC. The final trial had significant input toxicity to *H. azteca* and *C. dilutus*. The channel reduced toxicity to *H. azteca* and removed toxicity to *C. dilutus*. GAC filtration reduced *H. azteca* toxicity to an insignificant level. There was no input toxicity in the other three trials. The results demonstrate that a wetland treatment system coupled with GAC filtration can reduce pesticide concentrations, nutrients, suspended particles, and aquatic toxicity associated with agricultural runoff.

Monterey County, California, USA, contains a $4.4 billion/year agricultural industry that largely contributes to the nation’s produce supply (Monterey County Agricultural Commissioner 2019). The Salinas Valley encompasses much of the county’s agriculture and yields crops such as salad greens, strawberries, artichokes, and Brussels sprouts (University of California Cooperative Extension 2017). Pesticide use can greatly increase crop yields, which imposes an increased demand for irrigation (Cahn and Phillips 2019). While some growing operations utilize modern irrigation practices such as drip tape and time clocks to better control applications, a significant amount of pesticide-laden irrigation runoff still contributes to local stream flow (Dowd et al. 2008; Ippolito and Fait 2019; Kellogg et al. 2002; Phillips et al. 2012; Stout et al. 2018; Vymazal and Brezinova 2015).

The input of pesticides into aquatic environments can have detrimental effects on ecological communities, as research has revealed clear relationships between pesticide concentration and surface water toxicity (Anderson et al. 2003; Anderson et al. 2014; Antwi and Reddy 2015; Hunt et al. 2006; Sánchez-Bayo et al. 2016; Solomon 2010). This leads to many watersheds within central California being listed as impaired under the Clean Water Act Section 303(d) (California State Water Resources Control Board 2017). Numerous studies have explored mitigation strategies to address this, including the use of sedimentation ponds with floating aquatic plant vegetation, the presence of field-adjacent vegetation buffers, and vegetated drainage ditches, which can slow flow and promote pesticide-bound particle settling and plant sorption (Cahn and Phillips 2019; Hunt et al. 2008; Moore et al. 2008; Syversen and Bechmann 2004).

Constructed wetlands are another treatment approach for reducing pesticide concentrations. Natural wetlands provide many ecosystem functions, including water filtration (Mitsch and Gosselink 2000), which translate to the use of constructed wetlands in reducing surface water contamination, as well as nutrient loads and suspended solids. Constructed wetlands have been utilized globally in municipal and industrial effluent treatment processes and are growing in popularity to address nonpoint source pollution associated with agricultural irrigation (Díaz et al. 2012; Vymazal 2013). These systems’ efficacy is attributed to the creation of longer hydraulic residence times, which further facilitate sediment settling and vegetative treatment components, as well as increase the potential for breakdown processes such as hydrolysis, photolysis, and microbial metabolism (Budd et al. 2009; Hunt et al. 2007; Sherrard et al. 2004; Tu et al. 2018; Vymazal 2013). Wetland treatment efficiency can be influenced by the size of the wetland, water depth, flora type and density, soil type, and contaminant input rate. The associated variability in treatment can ultimately influence the persistence of pesticides with a diverse range of chemical properties within the wetland system (Blankenberg et al. 2006; Budd et al. 2011; Díaz et al. 2012; Gaullier et al. 2018; Gorito et al. 2017; Krone-Davis et al. 2013; Vymazal and Brezinova 2015).

The sole reliance on any one of these treatment approaches can have limitations. Past studies indicate that aquatic toxicity was primarily linked to pesticide chemical classes such as organochlorines and organophosphates (Anderson et al. 2003; Hunt et al. 2003; Hunt et al. 1999), whereas recent research shows toxicity linked to classes such as pyrethroids and neonicotinoids (Anderson et al. 2017; Deng et al. 2019; Epstein and Zhang 2014; Morrissey et al. 2015). An integrated treatment system can therefore be an appropriate means to reduce pesticide loading, provided it has components to treat hydrophobic pesticides, such as those belonging to the pyrethroid chemical class, as well as more soluble pesticides, such as neonicotinoids (Anderson et al. 2011).

The addition of carbon filtration to traditional vegetated treatment systems can increase the efficiency of pesticide removal. Granulated activated carbon (GAC) has been utilized for decades in industrial applications and has long been suggested for contaminated soil amendment and surface water treatment (Denyes et al. 2013; Johns et al. 1998; Kalmykova et al. 2014; Pryor et al. 1999). In bench-scale laboratory experiments, Voorhees et al. (2017) were able to completely remove environmentally relevant concentrations of the neonicotinoid imidacloprid from simulated flows representative of agricultural runoff. Phillips et al. (2017, 2021) found the use of a vegetated ditch coupled with installations of GAC filters was effective at reducing loads of the organophosphate chlorpyrifos, as well as the neonicotinoid imidacloprid and the pyrethroid permethrin in runoff from simulated agricultural irrigation events (Phillips et al. 2021).

In this study, the effectiveness of a constructed wetland treatment system combined with a GAC filtration installation was evaluated at reducing pesticide concentrations, nutrients, suspended particles, and aquatic toxicity associated with agricultural runoff. Additional vegetation treatment in the form of pennywort (*Hydrocotyle* spp.) was included in some trials. This combination of treatments served to address the complex mixture of contaminants found in the wetland’s source water, the Tembladero Slough. This water body is listed as impaired under the Clean Water Act Section 303(d) due to contaminants associated with agricultural runoff on the central coast of California, as well as urban runoff (California State Water Resources Control Board 2017).

## Methods

### Study Site and Monitoring

**Fig. 1:**
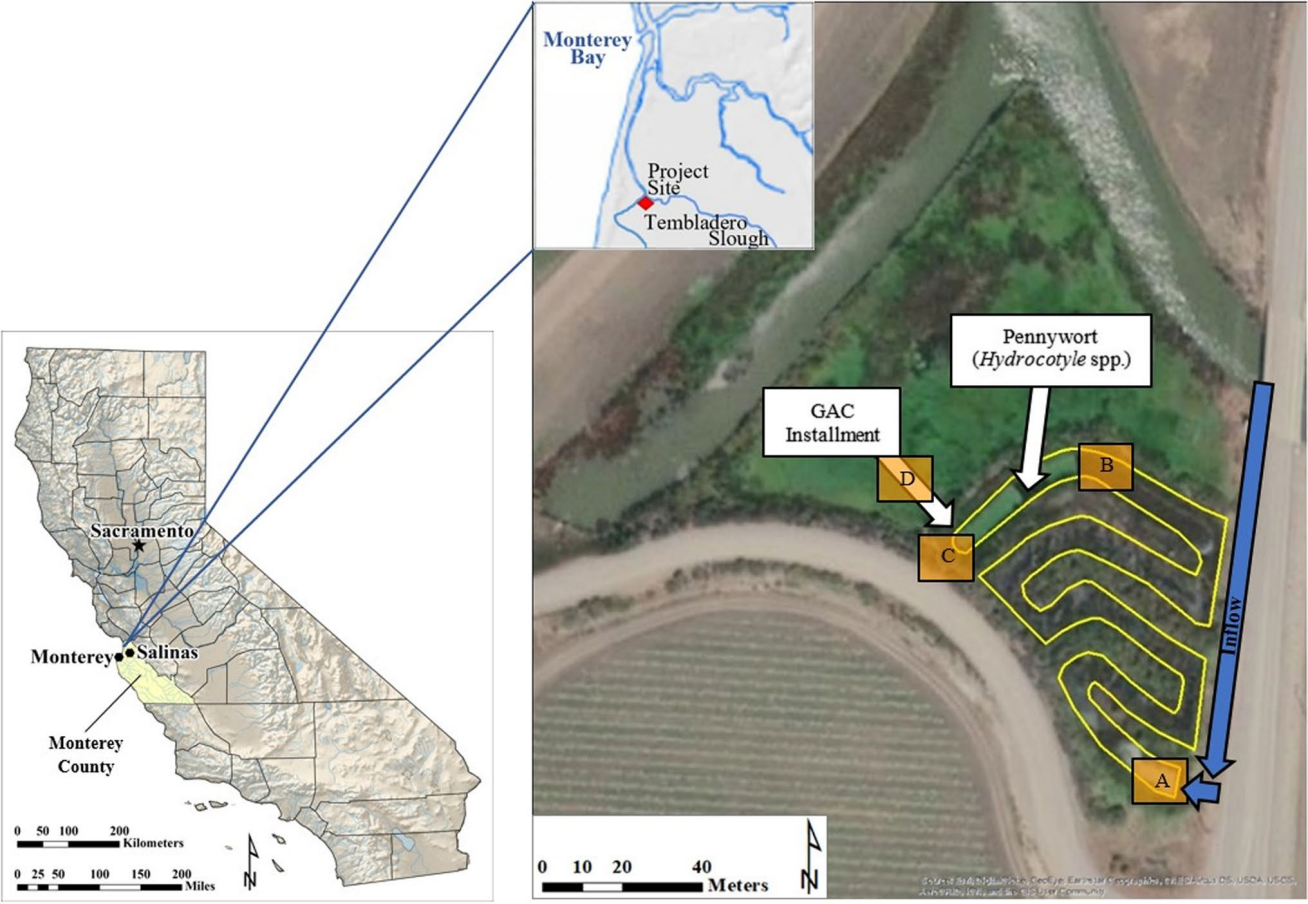
The Molera Road Experimental Treatment Wetland study site located in Monterey County, California, USA. Composite samples were collected at the wetland channel inflow (Station A), upstream of the pennywort (Station B, Trials 1 and 2), the channel outflow (Station C), and the outflow of the GAC filtration installation (Station D). Adapted from Hunt et al. (2007)

Five monitoring trials were conducted at the Molera Road Experimental Treatment Wetland during two agricultural growing seasons from September 2017 through December 2018 (Table 1). The fifth trial was conducted following a week of rain events to capture potentially increased contaminant loads from storm runoff. This constructed wetland, located just above the confluence of the Old Salinas River Channel and the Tembladero Slough (Fig. 1), resides in the Lower Salinas River Watershed (Monterey County, California, USA), which is comprised of approximately 380 km^2^ of predominately agricultural land, as well as some urban development (Miller 2014). Tembladero Slough water was pumped into a 285-m-long, 6.5-m-wide, and 0.3-m-deep sinuous channel dominated by cattails (*Typha* sp.) at a rate of approximately 360 L/min for 12 h/d during each monitoring trial, and 4 h/d during maintenance periods in between trials. Each trial occurred over a 24-h period, during which pumping was evenly staggered to disperse the inflow of water and not overwhelm the system. Pumping rates were increased from maintenance flows approximately 48 h prior to the start of each trial to allow for equilibrium within the system. Channel effluent drained onto approximately 0.6-ha of non-channelized marsh wetland and returned to the slough to eventually enter the Monterey Bay National Marine Sanctuary (Fig. 1) (Hunt et al. 2007).

**Table 1:** Dates and times of 24-h composite sampling

	Sampling Station	Start Date	Start Time	End Date	End Time
Trial 1	A	9/23/2017	09:30	9/24/2017	09:30
	B	9/25/2017	03:30	9/26/2017	03:30
	C	9/25/2017	09:30	9/26/2017	09:30
	D	9/25/2017	09:30	9/26/2017	09:30
Trial 2	A	7/15/2018	14:00	7/16/2018	14:00
	B	7/17/2018	06:00	7/18/2018	06:00
	C	7/17/2018	14:00	7/18/2018	14:00
	D	7/17/2018	14:00	7/18/2018	14:00
Trial 3	A	9/16/2018	09:00	9/17/2018	09:00
	C	9/18/2018	09:00	9/19/2018	09:00
	D	9/18/2018	09:00	9/19/2018	09:00
Trial 4	A	10/14/2018	09:00	10/15/2018	09:00
	C	10/16/2018	09:00	10/17/2018	09:00
	D	10/16/2018	09:00	10/17/2018	09:00
Trial 5	A	12/2/2018	09:00	12/3/2018	09:00
	C	12/4/2018	09:00	12/5/2018	09:00
	D	12/4/2018	09:00	12/5/2018	09:00

Station A is at the wetland channel inflow, Station B is upstream of the pennywort (Trials 1 and 2), Station C is the channel outflow, and Station D is the outflow of the GAC filtration installation

Previous studies have researched this wetland’s ability to reduce nutrients, suspended particles, and pesticide concentrations, particularly the more frequently detected organophosphate diazinon (Harris et al. 2007; Hunt et al. 2007; Miller 2014). In this study, modifications were made to optimize the treatment of current-use pesticides, which may not be removed by processes such as sedimentation and plant sorption due to a higher solubility (Phillips et al. 2017). A flow-through installation containing approximately 400 L of coconut/coal mix granulated activated carbon with an apparent density of 0.46 to 0.60 g/cm^3^ (GAC, Evoqua Water Technologies, Pittsburg, PA, USA) was placed at the outflow of the wetland channel to sorb more soluble pesticides. The GAC was not replaced during the five trials, although the design of the filtration installation evolved throughout the study. Resultantly, the GAC was removed prior to each change in design and was only actively used for approximately 6 months. During the first trial, GAC-filled geotextile Filtrexx Mesh™ socks (Filtrexx, Akron, OH, USA) were placed in a shallow wooden trough lined with Visqueen™ polyethylene sheeting. The GAC was removed from the system at the conclusion of Trial 1 while improvements were made for the second trial with the introduction of a fiberglass flow-through tank in which the GAC was loosely contained. To better ensure proper water movement and treatment efficiency, the installation was further modified for subsequent trials. The GAC was again removed following Trial 2 and placed into new Filtrexx Mesh™ socks for the remaining three trials. Additionally, a diffuser was added in the tank to evenly disperse the water flow through the carbon treatment.

The wetland channel was also modified by transplanting the floating aquatic plant pennywort (*Hydrocotyle* spp.) to approximately 20% of the distal end (Fig. 1). Pennywort can aid treatment by slowing water flow and providing additional surface area for sorption (Anderson et al. 2011; Hunt et al. 2008). This treatment was used only in the first two trials before it was deemed unsustainable due to grazing from local fauna, as well as a potential intolerance to elevated conductivity pulses from the tidally influenced Tembladero Slough.

Water samples were collected using Global Water automatic samplers (Xylem, Inc., College Station, TX, USA), which were stationed at the channel inflow (Station A), upstream of the pennywort in the first two trials (Station B), at the channel outflow (Station C), and at the outflow of the GAC filtration installation (Station D) (Fig. 1). At each sampling station, the automatic samplers were programed to collect 800 mL of sample every hour over a 24-h period. Composite samples were collected in 19-L glass carboys kept in the dark. The start of sampling was staggered from the inflow of the wetland channel toward the outflow in all trials to capture a 48-h hydraulic residence time, and to better evaluate potential contaminant reductions as water progressed through the system. Sampling at Station B, upstream of the pennywort treatment, started 42 h and 40 h after the start of sampling at Station A in Trial 1 and Trial 2, respectively. The sampling start times at Stations C and D, channel outflow and post-GAC, were not staggered (Table 1). The water moving through the GAC filtration installation appeared to have a short residence time, and it was assumed that collecting a composite sample over the 24-h sampling period would be sufficient in capturing additional treatment of channel water with GAC. Following each trial, samples were transferred to Thermo Scientific™ (Thermo Fisher Scientific, Waltham, MA, USA) certified amber glass bottles and maintained at 4 °C in the dark until chemical analyses and toxicity test initiation.

### Pesticide Chemistry and Statistical Analyses

Each composite sample was analyzed for 170 pesticides and degradates at the United States Geological Survey (USGS) California Water Science Center (Sacramento, California, USA), with one liter extracted for liquid chromatography tandem mass spectrometry (LC/MS/MS) analysis, and one liter for gas chromatography mass spectrometry (GC/MS). Samples were first filtered through 0.7-μm glass-fiber filters (Grade GF/F, Whatman, Piscataway, NJ, USA), with filter papers containing suspended sediments dried at room temperature overnight in the dark before being stored at – 20 °C until extraction and analysis with GC/MS. Detected pesticide concentrations from filter paper extracts were converted from ng/g to ng/L and included with the detected pesticides extracted from the filtered water samples analyzed with LC/MS/MS and GC/MS/MS, which were reported in ng/L. Detailed extraction procedure and instrumental analysis of the water samples analyzed with LC/MS/MS are fully described in Hladik and Calhoun (2012). The complete extraction procedure and GC/MS instrumental analysis are described in Hladik et al. (2008, 2009) and Hladik and McWayne (2012), respectively. Method detection limits (MDLs) for detected pesticide concentrations in water samples were validated in the previous work (Hladik and Calhoun 2012; Hladik et al. 2008) by using the procedure described in 40 CFR 136, Appendix B of United States Environmental Protection Agency (1992). MDLs for pesticides in suspended sediments filtered from water samples were validated in previous studies by Hladik et al. (2009) and Hladik and McWayne (2012). MDLs were used as quantitative reporting levels. As analytes can sometimes be identified at concentrations less than MDLs, concentrations of compounds detected below the MDLs were reported as estimates (De Parsia et al. 2018; Hladik and Calhoun 2012; Hladik and McWayne 2012; Hladik et al. 2008, 2009; Stout et al. 2018). All targeted analytes and methods of analysis are listed in the appendices section of the final project report of this study prepared for the California Department of Pesticide Regulation (McCalla et al. 2020).

Statistical significance (*α* = 0.05) of changes in pesticide concentrations across treatments with data from all trials was evaluated using several approaches. Two linear mixed effects models were used to assess changes in the logged pesticide concentrations across sampling stations (A, C, and D). Each model was implemented in the R package “lme4” (Bates et al. 2015; R Core Team 2019) and assumed normality. An analysis of the residuals plots indicated that a log transformation of the detected pesticide concentrations was sufficient for achieving normality (Zuur et al. 2009). In the first model, pesticides were grouped by assumed use: fungicide, herbicide, and insecticide. In addition to sampling station, trial and pesticide group were used as predictors of pesticide concentration, as well as interactions between station and trial and between pesticide group and trial. This was done to assess whether the change in pesticide concentration across sampling stations and the change in concentrations of different pesticide groups differed among trials. In the second model, pesticide chemical class was included instead of pesticide group to evaluate whether treatment effectiveness differed among classes. In both models, detected analytes were fitted as random effects to account for a lack of independence among measurements of pesticides from the same trial. In the first model, analyte random effects were nested within a random effect of pesticide chemical class to help account for variation.

Pairwise comparisons of logged pesticide concentrations between stations were used to evaluate the efficiency of each treatment component with the R package “emmeans” (Lenth 2021; R Core Team 2019), and *p*-values were adjusted using the Benjamini and Hochberg method for controlling the rate of false discovery (Benjamini and Hochberg 1995). Additionally, the impact of pesticide solubility, based on log *K*_ow_ values, on treatment efficacy was evaluated for both the wetland channel and GAC filtration component with linear regression models, where the detected analytes were the observations.

Statistical analyses in this study also incorporated non-detection data, or censored values, substituted with the MDLs in place of reporting limits under the guidance of the USGS California Water Science Center. For comparison of treatment efficiency within each trial, any observations of pesticides that had been detected at a measurable amount at one sampling station, but that were known to be less than the corresponding threshold value at another station, were then substituted with the appropriate MDL. This was done to avoid bias by deleting censored observations, to increase power, and provide confidence in parameter estimates (Helsel 2012).

### Aquatic Toxicity Tests

Toxicity tests were conducted following every trial with each 24-h composite sample at the University of California, Davis, Marine Pollution Studies Laboratory at Granite Canyon in Monterey, California, USA, with three invertebrate species that have varying sensitivities to agricultural pesticides. These tests included acute 96-h static renewal exposures with the cladoceran *Ceriodaphnia dubia* and the amphipod *Hyalella azteca*, as well as a chronic 10-d static renewal exposure with the dipteran *Chironomus dilutus*. All testing procedures followed modifications of United States Environmental Protection Agency methodology (Ingersoll et al. 2013; Kunz et al. 2017). Reference toxicant tests were conducted concurrently with every toxicity test to bracket the median lethal concentration (LC_50_), and to demonstrate suitability of test methodology. The reference toxicant tests were comprised of dilutions of reagent grade copper chloride for *C. dubia*, reagent grade cadmium chloride for *H. azteca*, and reagent grade potassium chloride for *C. dilutus*.

Toxicity data were evaluated using the Test for Significant Toxicity (TST). This statistical approach uses hypothesis testing to indicate whether the response in each sample is greater than or equal to a defined proportion of the control response (Denton et al. 2011; United States Environmental Protection Agency 2010b).

Dissolved oxygen, pH, and conductivity were measured at test initiation, following every renewal, and at test termination with an Accumet™ meter and appropriate electrodes (Thermo Fisher Scientific, Waltham, MA, USA). Un-ionized ammonia was measured at test initiation and termination using a Hach DR/2010 spectrophotometer (Hach, Loveland, CO, USA), and hardness and alkalinity (Hach) were measured only at test initiation. Composite water samples were also measured for nitrate and phosphate using a Hach DR/2010 spectrophotometer, as well as for turbidity with a Hach 2100P portable turbidimeter at test initiation (American Public Health Association et al. 1998). Water temperature was recorded with a continuous recording thermometer (Onset Computer Corporation, Pocasset, MA, USA), and additional daily temperatures were measured manually using a glass spirit thermometer.

## Results

### Pesticide Chemistry and Treatment Significance

Of the 170 targeted pesticides, 63 were detected across the five monitoring trials, encompassing 30 chemical classes, including some detections that were unclassified. All measured pesticide detections in each trial can be found in the appendices section of the final project report of this study prepared for the California Department of Pesticide Regulation (McCalla et al. 2020). Average percent changes in concentration within each treatment component were calculated by trial with all detected pesticides, as well as within three groups of assumed pesticide use: fungicide, herbicide, and insecticide (Table 2). The high percent increase in pesticide concentrations within the channel, from Stations A to C, during Trial 1 was mostly driven by the benzimidazole fungicide carbendazim. Treatment effectiveness seemed to vary across the trials, but concentrations of most pesticides decreased within the integrated system, from Stations A to D.

**Table 2:** Percent changes in pesticide concentrations

	Concentration range (ng/L)	2.6–9564	2.7–9564	2.7–652	2.6–1366
	Treatment Component	All pesticides % Change	Fungicide % Change	Herbicide % Change	Insecticide % Change
Trial 1	A–B	4	8	19	44
	B–C	127	198	− 6	− 3
	A–C	136	174	12	40
	C–D	− 69	− 79	− 17	− 29
	A–D	− 26	− 34	− 7	− 1.3
Trial 2	A–B	− 29	− 35	6	− 28
	B–C	7	15	15	− 6
	A–C	− 24	− 25	22	− 32
	C–D	− 90	− 92	− 85	− 88
	A–D	− 92	− 94	− 82	− 92
Trial 3	A–C	− 3	8	6	− 7
	C–D	− 70	− 69	− 68	− 72
	A–D	− 69	− 67	− 66	− 74
Trial 4	A–C	− 11	13	44	− 30
	C–D	− 24	− 23	− 18	− 27
	A–D	− 33	− 13	17	− 49
Trial 5	A–C	− 35	− 39	− 36	− 31
	C–D	− 10	6	− 22	− 24
	A–D	− 42	− 35	− 50	− 47

Stations A to B are from the channel inflow to pre-pennywort, B to C represent the pennywort treatment (Trials 1 and 2), A to C the entire wetland channel, C to D the GAC filtration, and A to D the integrated system. Negative and positive numbers indicate average percent concentration reduction and increase, respectively

#### Pennywort Treatment

The determination of statistically significant changes in pesticide concentrations was complicated by the inclusion of the pennywort treatment component in approximately 20% of the distal end of the wetland channel in only Trials 1 and 2. Three transplant efforts were conducted before the first trial, and by the end of Trial 2, the vegetation had been fully extracted by local fauna before it could become properly established.

An individual analysis of the pennywort treatment was first conducted to determine if it significantly affected pesticide concentrations in Trials 1 and 2. This linear mixed effects model included a fixed effect of Stations B and C, before and after exposure to the pennywort, a random effect of detected analytes nested in pesticide chemical class, and the log of pesticide concentrations as the response. The analysis concluded that there was no significant effect of the pennywort on pesticide concentrations (*F*(1, 122) = 0.379, *p* = 0.539). This finding allowed for the inclusion of Trials 1 and 2 in the system-wide analyses while excluding Station B (pre-pennywort).

#### Treatment Comparisons

Pairwise comparisons of estimated marginal means for the sampling stations were performed to evaluate the effectiveness of each treatment component. This analysis incorporated the data from all monitoring trials rather than conducting trial-specific pairwise t tests with smaller datasets to reduce type I and type II errors. The contrast of Stations A to C was used to assess treatment within the wetland channel, C to D the GAC filtration, and A to D within the integrated system. The results showed that there was overall significant treatment in both the channel (*p* = 0.009) and GAC filtration (*p* < 0.001). The estimate values, or average change in log concentrations between the stations, for the channel and GAC were 0.315 and 0.602, respectively, revealing that most of the system-wide treatment occurred within the GAC filtration component.

#### Treatment Effectiveness of Fungicides, Herbicides, and Insecticides

A linear mixed effects model was conducted to evaluate if there was a significant interaction between sampling stations, or treatment sections, and groups of assumed pesticide use: fungicide, herbicide, and insecticide. For this model, the fixed effects were the interactions between station and group and station and trial, the random effect was detected analytes nested in pesticide chemical class, and the response was the log of pesticide concentrations. The model produced four outputs, and the first revealed that there was a significant effect of sampling station on all pesticide concentrations (*F*(2, 570) = 94.0, *p* < 0.001), signifying that detected pesticide concentrations significantly changed as water moved through the system. There was also a significant difference in concentrations among the pesticide groups (*F*(2, 38) = 3.91, *p* = 0.028), indicating that the concentrations in some groups were significantly higher or lower than others. However, the model showed that there was no significant interaction between sampling station and pesticide group, revealing that there were no differences in treatment based on pesticide group (*F*(4, 570) = 0.529, *p* = 0.715). The model also showed that treatment effectiveness by pesticide group was variable among the trials (*F*(8, 570) = 14.9, *p* < 0.001). Although there was significant change in pesticide concentrations, pesticide group based on intended use had no influence on treatment effectiveness.

#### Treatment Effectiveness of Pesticide Chemical Classes

A second linear mixed effects model was conducted to determine treatment effectiveness of detected pesticide chemical classes, and whether treatment by class significantly differed between the wetland channel and GAC filtration components. This model included two fixed interaction effects of sampling station and pesticide class and station and trial and detected analytes as the random effect. There was a significant difference in pesticide concentrations as a result of treatment in the whole system (*F*(2, 512) = 96.3, *p* < 0.001), as well as a significant interaction between sampling station and trial (*F*(8, 512) = 15.3, *p* < 0.001). This revealed that treatment effectiveness of detected pesticides varied across the monitoring trials. There was also a significant difference in average concentrations between the detected chemical classes (*F*(31, 31) = 2.94, *p* = 0.002). However, there was no significant interaction between sampling station and pesticide class (*F*(62, 512) = 1.20, *p* = 0.150), indicating that there was no difference in treatment effectiveness across the pesticide chemical classes between the wetland channel and GAC treatment components.

#### Effects of Pesticide Solubility on Treatment Efficiency

**Fig. 2:**
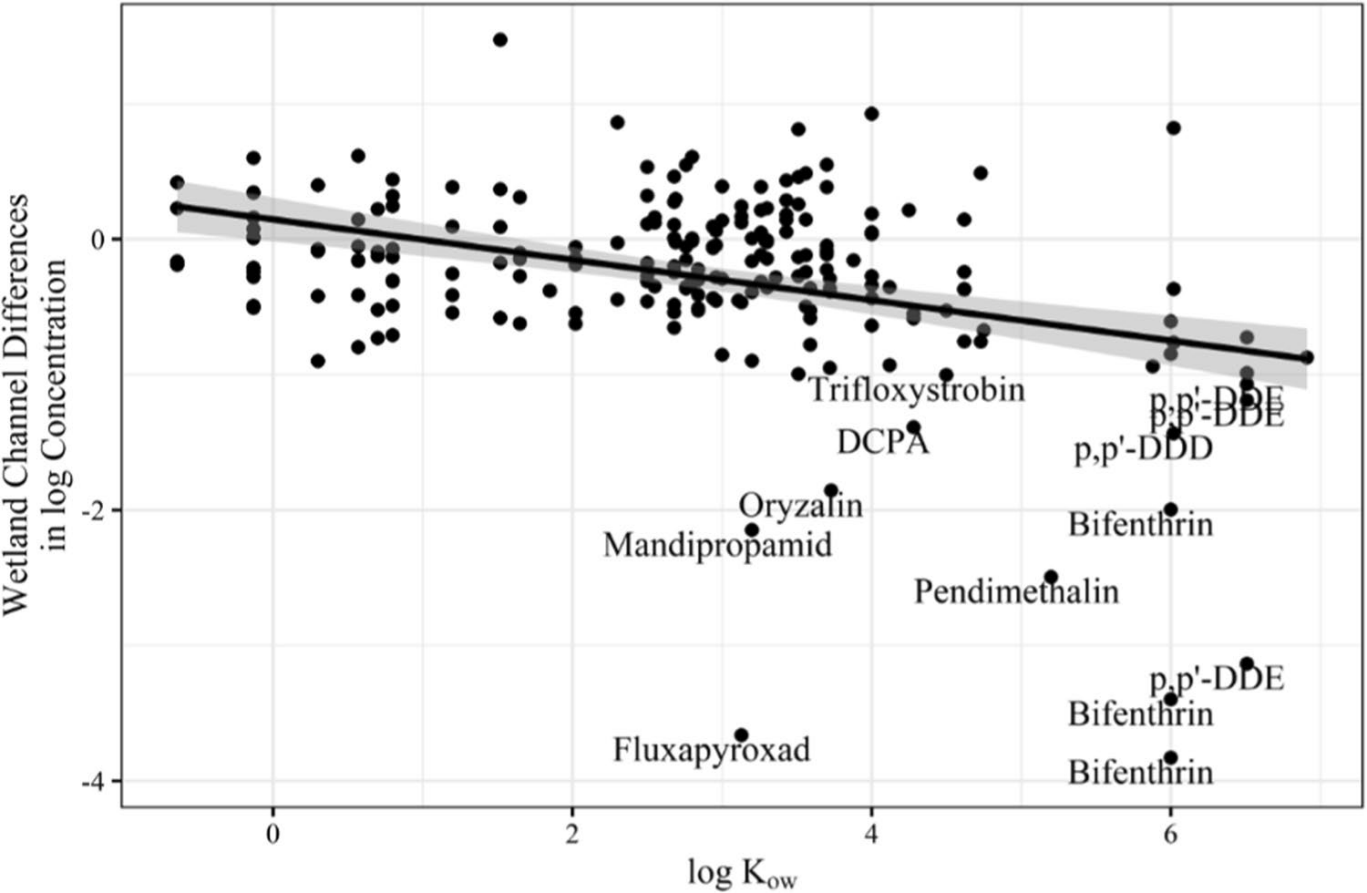
Difference in log-transformed pesticide concentrations across all trials within the wetland channel, between Stations A (channel inflow) and C (channel outflow), related to log K_ow_ values of detected pesticides

Considering there was no significant difference in treatment of detected chemical classes with the wetland channel and GAC filtration, the impact of pesticide solubility based on the octanol/water partition coefficient (log *K*_ow_) of each detected pesticide was investigated with a linear regression model for each treatment component. Pesticides with a low to moderate log *K*_ow_ value, ranging from approximately – 1 to + 4.5, are more soluble in aquatic environments, whereas those with high values, >  + 4.5, are more hydrophobic and readily bind to or associate with sediment and plant material (Chamberlain et al. 1996; Cumming and Rücker 2017; Finizio et al. 1997; Mackay et al. 1980; Meylan and Howard 1995). The model of the channel indicated a significant effect of solubility on the change in pesticide concentrations (*F*(1, 207) = 33.2, *p* < 0.001), which seemed to be driven by some of the less soluble pesticides with higher log *K*_ow_ values (Fig. 2). This trend was consistent across the trials as the model indicated no significant interaction between solubility and trial (*F*(4, 207) = 1.75, *p* = 0.141).

**Fig. 3:**
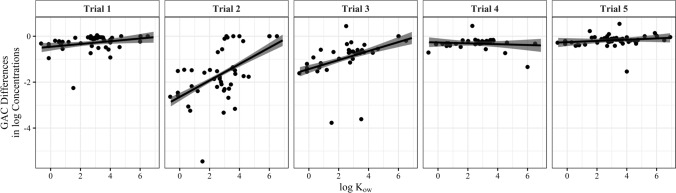
Difference in log-transformed pesticide concentrations by trial within the GAC filtration component, between Stations C (channel outflow) and D (post-GAC), related to log *K*_ow_ values of detected pesticides

The model analysis for the GAC filtration component also showed a significant effect of solubility on the difference in pesticide log concentrations (*F*(1, 207) = 25.3, *p* < 0.001). More soluble pesticides with low to moderate log *K*_ow_ values, approximately − 1 to + 4.5, exhibited greater differences in log concentrations before and after the carbon filtration system than less soluble compounds. However, there was a significant interaction between solubility and trial (*F*(4, 207) = 7.62, *p* < 0.001), revealing that this effect was not consistent across the trials (Fig. 3).

### Reduction of Nutrients

Composite samples from each sampling station were also measured for nitrate, phosphate, and turbidity at toxicity test initiation for every trial, except Trial 1 in which only turbidity was measured. On average, the integrated treatment system reduced nitrate concentrations by 61%, phosphate by 73%, and turbidity by 90%. The wetland channel, from Stations A to C, accounted for most of the average percent reductions of nutrients and suspended particles, with GAC filtration providing slight to moderate additional reductions in some trials (Table 3). As nutrients were not measured in Trial 1, an assessment of pennywort treatment effectiveness could not be completed. However, turbidity measurements increased by 71% after the pennywort in Trial 1 and decreased by 66% in Trial 2, indicating the low density of pennywort had a variable effect on particle reduction.

**Table 3:** Percent changes in nutrient concentrations and turbidity between sampling stations by trial

	Treatment Component	Nitrate % Change	Phosphate % Change	Turbidity % Change
Trial 1	A–B	NA	NA	− 95
	B–C	NA	NA	71
	A–C	NA	NA	− 92
	C–D	NA	NA	− 36
	A–D	NA	NA	− 95
Trial 2	A–B	− 53	− 50	− 57
	B–C	4	− 19	− 66
	A–C	− 51	− 60	− 85
	C–D	− 4	− 12	− 59
	A–D	− 52	− 64	− 94
Trial 3	A–C	− 55	− 64	− 79
	C–D	0	− 19	− 41
	A–D	− 55	− 70	− 88
Trial 4	A–C	− 62	− 86	− 88
	C–D	− 4	11	− 18
	A–D	− 63	− 84	− 90
Trial 5	A–C	− 75	− 77	− 84
	C–D	6	14	− 2
	A–D	− 74	− 74	− 84

Stations A to B are from the channel inflow to pre-pennywort, B to C represent the pennywort treatment (Trials 1 and 2), A to C the entire wetland channel, C to D the GAC filtration, and A to D the integrated system. Negative and positive numbers indicate percent reduction and increase, respectively. NA is not analyzed

### Reduction of Aquatic Toxicity

All aquatic toxicity tests met test acceptability criteria, and water quality parameters were within acceptable limits. All accompanying reference toxicant tests produced acceptable results, indicating that test organisms responded to the positive controls in a manner consistent with previous tests. There was significant input toxicity to the daphnid *Ceriodaphnia dubia* and the amphipod *Hyalella azteca* in Trial 1. Toxicity to *C. dubia* persisted throughout the treatment system, including post-GAC filtration, whereas toxicity to *H. azteca* was removed by the channel. However, there was residual significant toxicity to *H. azteca* at Station D following the GAC filtration. There was no significant toxicity at the inflow of the channel or at other sampling stations in Trials 2–4. Trial 5 was conducted after several rain events, and there was significant input toxicity to *H. azteca* and the dipteran *Chironomus dilutus*. Amphipod toxicity was reduced by the channel, but not eliminated, whereas the channel removed toxicity to *C. dilutus*. GAC filtration reduced amphipod toxicity to an insignificant level (Table 4).

**Table 4:** Average percent survival for 96-h *Ceriodaphnia dubia*, 96-h *Hyalella azteca*, and 10-d *Chironomus dilutus* toxicity tests, and average ash-free dry weight as an indication of growth for the *C. dilutus* tests

	Sampling Station	*C. dubia* % Survival	*H. azteca* % Survival	*C. dilutus* % Survival	*C. dilutus* Growth (mg)
Trial 1	A	**60**	**34﻿**	100	3.67
	B	92	100	100	1.56
	C	**64**	100	100	3.32
	D	**36**	**72﻿**	98	3.73
	Control	100	100	100	2.45
Trial 2	A	100	100	98	1.02
	B	100	98	98	1.18
	C	100	100	98	1.02
	D	96	96	100	1.12
	Control	100	100	96	1.71
Trial 3	A	100	86	98	3.19
	C	92	100	100	2.27
	D	92	98	100	2.79
	Control	96	96	96	2.80
Trial 4	A	100	98	100	5.89
	C	96	100	98	5.42
	D	96	100	98	5.48
	Control	96	100	94	7.29
Trial 5	A	100	**0﻿**	**﻿6**	**﻿0.02**
	C	96	**﻿68**	85	5.13
	D	92	80	94	5.34
	Control	96	96	94	4.60

Bold indicates significant toxicity. Station A is at the wetland channel inflow, Station B is upstream of the pennywort (Trials 1 and 2), Station C is the channel outflow, and Station D is the outflow of the GAC filtration installation

## Discussion

A previous study found that the Molera Road Experimental Treatment Wetland promotes particle settlement, plant sorption, hydrolysis, photolysis, and microbial breakdown (Hunt et al. 2007). The presence of densely rooted cattails (*Typha* sp.), a common macrophyte found in North American wetlands, along the channel’s margins can further facilitate in slowing water movement and the adsorption and uptake of pesticides (Main et al. 2017; Moore et al. 2017; Stang et al. 2016). Hunt et al. (2007) found that the system reduced pesticide concentrations, particularly the frequently detected organophosphate insecticide diazinon, and associated aquatic toxicity. Although organophosphates were not detected in this study, there were significant changes in detected pesticide concentrations within the wetland channel, predominately those with more hydrophobic properties (Fig. 2).

The entire system decreased pesticide concentrations over a wide range of solubilities with the inclusion of the GAC filtration treatment, which reduced average percent concentrations of detected analytes in all trials (Table 2), particularly the more soluble pesticides (Fig. 3). The variability in the efficiency of this treatment system could be the result of several factors, including the adsorption capacity of the activated carbon, as it was not replaced during the study. While the lifespan of GAC being utilized in a watershed treatment system such as a constructed wetland is unclear, continual filtration of agricultural and urban drainage during approximately 6 months of the study period could account for some of the observed reduction in GAC filtration efficiency following Trial 2. Newcombe et al. (1993) described mechanisms of carbon regeneration and found that over time high loads of organic material in source water have been shown to reduce the life of carbon filtration components.

The evolving design of the GAC filtration system throughout the study could have also contributed to inconsistent treatment. In Trial 1, which utilized the GAC in mesh sleeves but had a fair amount of bypass in a shallower filter design, carbon filtration (Stations C to D) provided a 69% reduction in pesticide concentrations, which had substantially increased within the channel, before and after the pennywort (Stations B to C). The GAC was removed from the system at the conclusion of Trial 1 while improvements were made for the second trial. GAC exhibited the best performance in Trail 2, in which the carbon was loose within a fiberglass tank, with a 90% concentration reduction. The GAC was again removed following Trial 2 and placed into new Filtrexx Mesh™ socks for Trials 3–5, as it was assumed this configuration would perform as well, or better, than loose carbon in addition to being easier to manage. However, over the course of these last three trials, GAC treatment efficiency decreased from 70 to 10%. Interestingly, percent reductions within the wetland channel simultaneously improved from 3 to 35% during these trials (Table 2).

Although there were certainly variable concentrations of pesticides exhibiting different physical–chemical properties entering the Tembladero Slough during local applications, it is important to remember that this waterbody receives runoff from within a watershed of approximately 380 km^2^ (Miller 2014) and is continuously a complex mixture of contaminants. There was no clear difference in analyte detections between Trials 1–4 across the two growing seasons. Trial 5, however, followed recent rains and exhibited several additional pesticide detections, as well as higher input concentrations of most of the previously detected pesticides, likely the result of elevated particle loading related to the storm events. This trial had the lowest percent concentration reduction with GAC filtration, and most reductions occurred within the wetland channel (Table 2), perhaps through pesticide-bound particles settling and plant sorption.

Increases in some pesticide concentrations within the system could have been the result of inadequately capturing the pulse of water within the staggered 24-h composite at each sampling station. In Trials 1–4, for example, the benzimidazole fungicide carbendazim was detected at a higher concentration at the outflow of the channel (Station C) than that measured at the inflow (Station A). Within these trials, GAC filtration reduced the elevated concentration to less than that measured at the channel inflow. However, in Trial 5 this fungicide was reduced within the wetland channel but exhibited an increase in concentration following GAC filtration. Similarly, the strobilurin fungicide azoxystrobin was also detected at higher concentrations at the outflow of the channel in Trials 1–4. GAC filtration provided reductions in three of the trials, but in only one was the analyte reduced to a concentration less than that observed at the channel inflow. The benzamide herbicide propyzamide was also detected in increasing concentrations within the wetland channel during all five trials, and while GAC provided reductions in each trial, concentrations were reduced to below channel inflow measurements in only three of them. The pennywort may have also introduced pesticide residue in Trials 1 and 2, as the source of the plant material was a nearby agricultural drainage, and some pesticide concentrations increased from Stations B to C, pre- and post-pennywort.

There are many variables to consider when assessing the fluctuating efficiency of constructed wetland treatment systems, but the data reinforce the effectiveness of constructed wetlands at reducing concentrations of hydrophobic pesticides, while outlining the need for an additional treatment component to address the more soluble pesticides also found in modern agricultural runoff. The additional use of toxicity testing is necessary when monitoring treatment effectiveness because toxicity is often caused by complex mixtures of pesticides in which non-toxic concentrations of detected analytes may have additive or synergistic effects. Organisms will respond to the bioavailable fraction of multiple contaminants, and the resolution of toxicity monitoring is increased through the use of multiple species with varying sensitivities (Anderson et al. 2017).

In Trial 5, toxicity was removed for *C. dilutus* and reduced for *H. azteca* in the channel, which was further reduced with GAC filtration (Table 4). Current-use pesticides likely to contribute to toxicity include the pyrethroid and neonicotinoid insecticides. The only pyrethroid detected throughout the study was bifenthrin, which was measured at the highest concentration at the channel inflow (Station A) in Trial 5, and was approximately 4 times the median lethal concentration (LC_50_) for *H. azteca* (9.3 ng/L 96-h LC_50_, Anderson et al. 2006) and about 1.6 times the LC_50_ value for *C. dilutus* (23 ng/L 10-d LC_50_, Ding et al. 2012). As water moved through the integrated system, bifenthrin was reduced by approximately 43%. A range of neonicotinoids were also detected at the channel inflow during Trial 5 but did not exceed known LC_50_ values. All neonicotinoid concentrations were decreased within the channel, from Stations A to C, with further reductions following GAC filtration.

The causes of toxicity to *C. dubia* and *H. azteca* in Trial 1 are less clear, and it is hard to draw conclusions from the complex mixture of detected pesticides. No insecticides were detected at higher input concentrations than in Trial 5, and only several fungicides and herbicides were detected at higher concentrations in this trial. The fungicide carbendazim was detected at far greater concentrations than any other pesticide in Trial 1, exceeding 9000 ng/L at the outflow of the channel. Carbendazim is moderately toxic to many aquatic organisms (International Union of Pure and Applied Chemistry 2019), although the literature is lacking in the direct effects of this analyte on *C. dubia* and *H. azteca*. The daphnids *Daphnia pulex* and *Daphnia magna* have reported LC_50_ values of 136,100 ng/L for 48 h (Encina et al. 2017) and 270,000 ng/L for 96 h (Verschueren 2001), respectively. Juveniles of the freshwater amphipod *Gammarus pulex* have been observed to have a 48-h LC_50_ value of 77,000 ng/L and a 96-h LC_50_ of 55,000 ng/L (Van Wijngaarden et al. 1998). Regardless, following toxicity at the channel inflow (Station A) in Trial 1, there was no observed toxicity within the channel just before the pennywort (Station B) to both *C. dubia* and *H. azteca*. Toxicity returned after the pennywort at the channel outflow (Station C) to *C. dubia*, and to both organisms following GAC filtration (Station D). However, all measured pesticide concentrations post-GAC were less than the measured values in the channel before the pennywort. It is therefore presumed that elevated conductivity pulses contributed to daphnid toxicity, as the Tembladero Slough is tidally influenced, and post-GAC toxicity to daphnids and amphipods was attributed to the materials used to construct the carbon installation in this first trial, such as the polyethylene trough lining. Additionally, this study targeted a finite number of analytes and there may have been others not measured that could have contributed to toxicity.

## Conclusions

The results of this study demonstrated that the Molera Road Experimental Treatment Wetland coupled with GAC filtration provided significant pesticide treatment, and reduced toxicity, nutrients, and suspended particles associated with agricultural runoff. These reductions were likely the result of numerous factors, including a controlled hydraulic residence time allowing for reduced flow, and therefore, an increased potential for sediment settling, plant sorption, hydrolysis, photolysis, and microbial metabolism. The observed variability in treatment was likely influenced by factors such as the complex mixture of pesticides exhibiting different physical–chemical properties entering the Tembladero Slough, changing environmental parameters, and the evolving design of the GAC filtration component.

Further research with this integrated wetland treatment system would be beneficial in better understanding the effectiveness of treating nonpoint source agricultural runoff. For instance, trials could be conducted with varying hydraulic residence times, vegetation pesticide removal could be quantitatively measured, and sediment chemical analyses and toxicity tests could be periodically carried out to evaluate pesticide accumulation within the system. This study has also provided a foundation to better characterize the operational lifespan of GAC being utilized in a watershed treatment system, particularly when used as a final treatment component following a presumed reduction of organic material and suspended sediment. More field trials should be conducted to better understand the loading capacity of GAC under various environmental conditions. Additionally, continued investigations and evaluations could be conducted to identify other treatment methods for an integrated system. For example, biochar could be a more cost-effective and comparable alternative to GAC.

## Data Availability

All data generated and analyzed during this study are included in a final report written for the California Department of Pesticide Regulation, which is accessible online: https://www.cdpr.ca.gov/docs/emon/surfwtr/contracts/tembladero_final_report.pdf.

## Code Availability

The R code used for statistical analyses can be provided upon request from the corresponding author.
